# Effect of Treadmill Training Interventions on Spatiotemporal Gait Parameters in Older Adults with Neurological Disorders: Systematic Review and Meta-Analysis of Randomized Controlled Trials

**DOI:** 10.3390/ijerph19052824

**Published:** 2022-02-28

**Authors:** Alka Bishnoi, Rachel Lee, Yang Hu, Jeannette R. Mahoney, Manuel E. Hernandez

**Affiliations:** 1 Department of Kinesiology and Community Health, University of Illinois at Urbana-Champaign, Urbana, IL 61801, USA; abishn2@illinois.edu (A.B.); yangh3@illinois.edu (Y.H.); 2 Department of Solid Organ Transplant, University of Chicago Medical Center, Chicago, IL 60637, USA; rjlee5@illinois.edu; 3 The Saul R. Korey Department of Neurology, Albert Einstein College of Medicine, Bronx, NY 10461, USA; jeannette.mahoney@einsteinmed.edu

**Keywords:** treadmill, intervention, gait, neurological disorders

## Abstract

**Objective:** Treadmill interventions have been shown to promote ‘normal’ walking patterns, as they facilitate the proper movement and timing of the lower limbs. However, prior reviews have not examined which intervention provides the most effective treatment of specific gait impairments in neurological populations. The objective of this systematic review was to review and quantify the changes in gait after treadmill interventions in adults with neurological disorders. **Data Sources:** A keyword search was performed in four databases: PubMed, CINAHL, Scopus, and Web of Science (January 2000–December 2021). We performed the search algorithm including all possible combinations of keywords. Full-text articles were examined further using forward/backward search methods. **Study Selection:** Studies were thoroughly screened using the following inclusion criteria: study design: Randomized Controlled Trial (RCT); adults ≥55 years old with a neurological disorder; treadmill intervention; spatiotemporal gait characteristics; and language: English. **Data Extraction:** A standardized data extraction form was used to collect the following methodological outcome variables from each of the included studies: author, year, population, age, sample size, and spatiotemporal gait parameters including stride length, stride time, step length, step width, step time, stance time, swing time, single support time, double support time, or cadence. **Data Synthesis:** We found a total of 32 studies to be included in our systematic review through keyword search, out of which 19 studies included adults with stroke and 13 studies included adults with PD. We included 22 out of 32 studies in our meta-analysis that examined gait in adults with neurological disorders, which only yielded studies including Parkinson’s disease (PD) and stroke patients. A meta-analysis was performed among trials presenting with similar characteristics, including study population and outcome measure. If heterogeneity was >50% (denoted by I^2^), random plot analysis was used, otherwise, a fixed plot analysis was performed. All analyses used effect sizes and standard errors and a *p* < 0.05 threshold was considered statistically significant (denoted by *). Overall, the effect of treadmill intervention on cadence (z = 6.24 *, I^2^ = 11.5%) and step length (z = 2.25 *, I^2^ = 74.3%) in adults with stroke was significant. We also found a significant effect of treadmill intervention on paretic step length (z = 2.34 *, I^2^ = 0%) and stride length (z = 6.09 *, I^2^ = 45.5%). For the active control group, including adults with PD, we found that overground physical therapy training had the largest effect on step width (z = −3.75 *, I^2^ = 0%). Additionally, for PD adults in treadmill intervention studies, we found the largest significant effect was on step length (z = 2.73 *, I^2^ = 74.2%) and stride length (z = −2.54 *, I^2^ = 96.8%). **Conclusion:** Treadmill intervention with sensory stimulation and body weight support treadmill training were shown to have the largest effect on step length in adults with PD and stroke.

## 1. Introduction

In the United States, the number of adults ≥65 years of age is estimated to increase from 53 million in 2018 to 88 million in 2050. As this population increases in size, the financial burden on the healthcare system will also increase, and effective preventive and/or therapeutic approaches will be desperately needed to help diminish the increased burden associated with the increase in the number of older adults. Among the various age-related diseases, neurological disorders with or without concomitant cognitive decline are particularly relevant given their adverse impact on people’s quality of life [1]. Neurological disorders can have detrimental effects on gait and mobility, as they may increase the risk of falls and disability [2], leading individuals to maintain a sedentary lifestyle and have an increased fear of falling. However, improvements in walking ability provide a positive impact on the quality of life and health of older individuals, particularly as daily walking significantly reduces the risk of cardiovascular disease, osteoporosis, diabetes, and other chronic diseases in this aging population [3].

Stroke, one of the most common neurological disorders, is a leading cause of long-term disability in older adults. Brain damage due to stroke can lead to symptoms such as cognitive and motor impairments including pain, paralysis, poor balance, spasticity, muscle weakness, and ineffective gait patterns [4]. More than 80% of post-stroke survivors suffer from chronic walking dysfunction [5]. Adults with stroke are prone to injuries leading to falls and often require rigorous rehabilitation during the subacute and chronic phases. The goal of early stroke rehabilitation is to restore the ability to perform activities of daily living (ADL) such as walking, feeding, or toileting. In particular, regaining normal walking function is one of the most important concerns for people who have suffered a stroke and results in a significant amount of time spent focusing on re-learning how to walk [6]. Studies have shown that patients who partake in early intensive rehabilitation have better outcomes, especially in regard to regaining independent ambulation [7]. However, there is an extensive gait rehabilitation literature which makes it difficult for clinicians to choose optimally effective treatment plans.

Another common neurological disorder is Parkinson’s disease (PD). PD is a common neurodegenerative disorder that occurs in about 1% of adults over the age of 60 years old [8]. Adults with PD suffer from impaired basal ganglia function, leading to disturbances in gait and balance, to name a few. Major motor impairments include bradykinesia (slowness in movement), postural instability, rigidity, and resting tremor. Individuals with PD often have trouble picking their feet up while walking which leads to taking small, shuffling steps known as “Parkinsonian gait”. This type of gait impairment is associated with increased falls and can negatively impact the quality of life [9]. Like adults with stroke, adults with PD also undergo comprehensive rehabilitation programs to improve walking ability but often face difficulty re-gaining normal gait patterns.

Adults living with these conditions must undergo sustainable forms of short-term and long-term rehabilitation to regain function and perform the activities of daily living. Many studies have demonstrated that treadmill training is a common approach for improving mobility and gait. Treadmill training (TT) has been shown to promote ‘normal’ walking patterns, as it facilitates the proper movement and timing of the lower limbs, thus eliminating the need for compensatory gait mechanisms [10]. It can also improve spatiotemporal gait parameters such as stride length, swing time, and cadence. This systematic review focused on studies including treadmill intervention effects on spatiotemporal gait parameters of people with neurological disorders. Types of treadmill interventions were body weight support (BWS) TT, and TT with sensory cues or biofeedback. Although TT is a popular rehabilitation activity, there is an insufficient literature regarding its effectiveness in improving gait parameters in patients with neurological disorders [5]. Previous reviewers have not undertaken a quantitative synthesis of intervention effects on spatiotemporal gait parameters in common neurological populations [11]. If they carried out a quantitative synthesis, then it was not carried out on all gait parameters [5], and they also did not compare interventions in one neurological population to another to find out the best suitable intervention for the specific gait impairment [12].

Given the fact that TT is a widely accepted method of gait training, the purpose of this study was to investigate the effectiveness of TT in improving spatiotemporal gait parameters in older adults with neurological disorders. To date, there is no systematic review and meta-analysis which has been directed to summarize what is known from randomized controlled trials about the effectiveness of therapeutic interventions to address walking concerns associated with spatiotemporal gait characteristics among individuals with neurologic diseases. As such, it is difficult for physical therapists to provide the most effective, up-to-date, specific evidence-based intervention to individuals with neurologic diseases presenting with specific gait impairment. This systematic review aimed to: (1) quantify the effect of different treadmill interventions on spatiotemporal gait parameters of older adults with neurological disorders; and (2) evaluate each randomized controlled trial (RCT) based on the quality assessment criteria. While we tried to be inclusive of neurological disorders in this study, after applying the inclusion criteria, the two main neurological disorders were stroke and PD.

## 2. Materials and Methods

### 2.1. Study Selection Criteria

Studies that met all the following criteria were included in the review: (1) study design: randomized controlled trial; (2) year: published after 2000; (3) population: older adults ≥55 years old with a neurological disorder; (4) intervention: any type of treadmill intervention; (5) main outcome measures: spatial and temporal gait characteristics including stride length, stride time, step length, step width, step time, stance time, swing time, single support time, double support time, or cadence; and (6) publication language: English. Studies were excluded from the review if they contained one or more of the following exclusion criteria: (1) non-randomized controlled trials; (2) studies published before 2000; (3) population under the age of 55 years; (4) experimental groups contained only healthy older adults; and (5) studies that did not include any spatial or temporal gait characteristics.

### 2.2. Search Strategy

The systematic review and meta-analysis described in the Preferred Reporting Items for Systematic Reviews and Meta-analysis process was adopted to guide the review process. A keyword search was performed in 4 databases: PubMed, Cumulative Index to Nursing and Allied Health Literature (CINAHL), Scopus, and Web of Science. The search retrieved articles published from January 2000 to December 2021. The search algorithm was formulated to include all possible combinations of keywords from the following 5 groups: (1) walk OR ambulatory OR mobility OR gait; (2) variability OR complexity OR unsteadiness OR inconsis* OR stability OR equilibrium OR dynamics OR balance OR ataxia; (3) “neurological disorder” OR “neurological pathology” OR “multiple sclerosis” OR “Parkinson’s disease” OR “Huntington’s disease” OR ALS OR “cerebellar ataxia” OR Alzheimer OR stroke; (4) intervention OR therapy OR treatment OR “best practices”; (5) older adults OR elderly OR aged OR elder OR older OR senior OR geriatric. Certain keywords were made to be excluded, such as musculoskeletal OR posture OR postural OR animal OR robot OR amputee OR trunk OR knee OR hip OR freezing of gait OR spasticity OR heart OR blood OR cardiac. The search algorithm for each database can be found in Appendix A.

Titles and abstracts of the articles identified through the keyword search were screened for the study selection criteria. Two reviewers (A.B. and R.L.) independently performed title and abstract screening to determine eligibility. Interrater agreement was determined by Intraclass correlation coefficient value and authors showed a moderate correlation (ICC = 0.69). Any disagreements were resolved through discussion. Full-text articles were obtained after both authors agreed that the potential article satisfied all study selection criteria. A cited reference search (i.e., forward reference search) and reference list search (i.e., backward reference search) were performed based on the articles meeting all inclusion criteria identified from the keyword search. The articles that resulted from the forward and backward reference search were also screened and evaluated using the same study selection criteria. Three layers of forward and backward reference searches were completed until no additional relevant articles met the inclusion criteria.

### 2.3. Data Extraction

A standardized data extraction form was used to collect the following methodological outcome variables from each of the included studies: author(s), publication year, population, age, sample size, and outcome measures. Outcome measures include spatial gait characteristics (stride length, step length, step width) in centimeters, temporal gait characteristics including single-limb support time, double limb support time, cadence, stride cycle in either percentage or time (seconds) of the gait cycle, and cadence in steps/minute. If the characteristics were not provided in desired units, necessary conversions were performed.

For treadmill training groups in our study, if the study had a pure treadmill training (TT) group with an active control group involving conventional PT, or overground walking, then our pure TT group was considered the intervention group. However, if in the study there was a group involving TT with either sensory feedback, perturbation, or bodyweight support, then that was considered the intervention group, and the pure TT was considered the active control group.

### 2.4. Quantitative Data Synthesis

For research that utilized the same population in more than one study, meta-analysis was performed to estimate the pooled effect size (pre-intervention mean—post-intervention mean) and the standard error of specific spatiotemporal gait parameters under specific therapeutic intervention for a specific neurological population. The equation used was:

$$\sqrt{({\left(SDpre\right)}^{2}+{\left(SDpost\right)}^{2})/n}$$

Secondary analysis was performed to examine the effect of dosage (times × week = minutes) and age on cadence after pure TT and the effect of body weight support load and dosage on step length after body weight support TT in a specific neurological population. Study heterogeneity was assessed by using the I^2^ index. The level of heterogeneity represented by I^2^ was interpreted as modest (I^2^ < 25%), moderate (25% < I^2^ < 50%), substantial (50% < I^2^ < 75%) or considerable (I^2^ > 75%). A fixed model was estimated when modest to moderate heterogeneity was present, and a random-effect model was estimated when substantial to considerable heterogeneity was present. Publication bias was visually inspected using funnel plots and empirically tested using Egger’s tests. All statistical analyses were conducted using Stata 14.2 SE version (StataCorp, College Station, TX, USA). All analyses included 2-sided *t*-tests, where *p*-values < 0.05 were considered statistically significant.

### 2.5. Study Quality Assessment

We used the National Institutes of Health’s (NIH) study quality assessment tool for intervention studies to assess the quality of all included studies [13]. The following criteria questions were used: (1) Was the study a randomized controlled trial? (2) Was the study population specified and defined? (3) Was sample size justification, power description, or variance and effect estimates provided? (4) Was there an active control group? (5) Was the treatment assessor or participant blinded? (6) Were the study groups baseline matched? (7) Were the exposure measures (independent variables) clearly defined, valid, reliable, and implemented consistently across all study participants? (8) Were the outcome measures (dependent variables) clearly defined, valid, reliable, and implemented consistently across all study participants? (9) Were potential confounding variables measured and adjusted statistically for their impact on the relationship between exposure(s) and outcome(s)? (10) Were the gait cycle parameters clearly defined and uniformly applied to all participants? Each study was scored on each of these criteria ranging from a score of 0 to 2, depending on whether the criterion was unmentioned or unmet (0), partially met (1), or completely met (2); therefore, the highest possible total study score was 20 and the lowest was 0. The study quality assessment measured the strength of the articles but did not determine whether the study was included in the current meta-analysis.

## 3. Results

### 3.1. Study Selection

**Figure 1:**
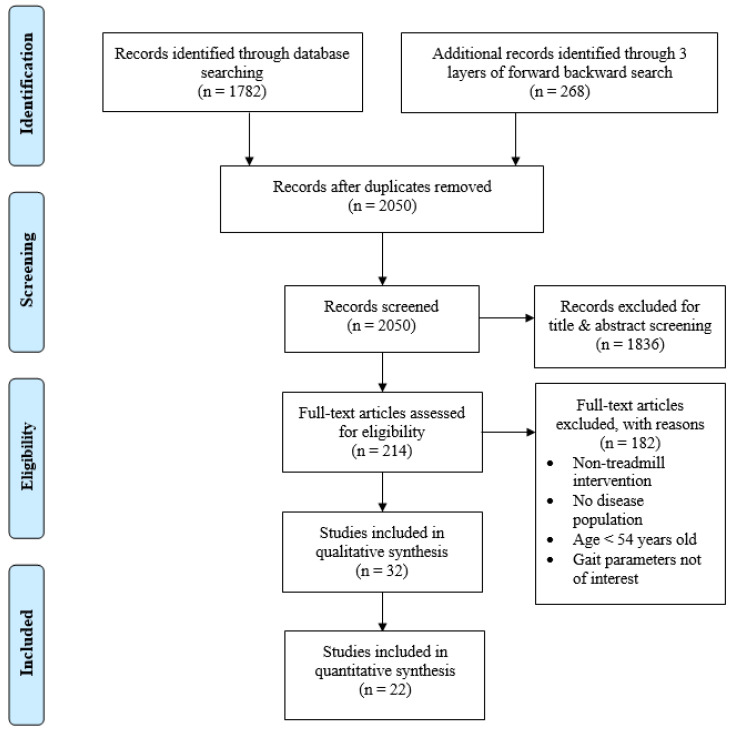
Study selection flow chart.

As illustrated in Figure 1, a total of 2050 unduplicated articles were identified through keyword and reference searches, and 1836 articles were excluded from the review after the title and abstract screening. Of the 214 articles subjected to full-text screening, 182 were excluded due to the reasons outlined in Figure 1. The remaining 32 articles [14,15,16,17,18,19,20,21,22,23,24,25,26,27,28,29,30,31,32,33,34,35,36,37,38,39,40,41,42,43,44,45] were included in qualitative synthesis for the review.

### 3.2. Basic Characteristics of Selected Studies

Basic characteristics for the age and sample size of all included studies are summarized in Table 1 by study. Among the final list of studies used for the review, the two disease populations mentioned are stroke and PD. Nineteen studies contain adults with history of stroke [14,15,16,17,18,19,20,21,22,23,24,25,26,27,28,29,30,31,32] and 13 studies contain adults with Parkinson’s disease [33,34,35,36,37,38,39,40,41,42,43,44,45]. When examining the studies that included a stroke population, 55.5% of the studies contained adults with chronic stroke [14,15,16,18,19,20,21,27,30,31,32] and 27.8% of the studies contained adults with acute or subacute stroke [23,24,25,27,29]. Eleven studies included adults with both hemorrhagic and ischemic stroke [14,18,19,20,21,24,27,29,30,31,32]. One study contained adults with only ischemic stroke [16]. Seven studies did not mention the cause of stroke [15,17,22,23,25,26,28]. Three studies did not provide any information regarding the duration or cause of stroke [17,22,26]. As for the studies containing adults with PD, 12 out of 13 studies reported the inclusion of patients in Stage 1–3.5 on the Hoehn and Yahr scale [33,34,35,36,37,38,39,40,41,43,44,45] and only one study included patients in Stage 4 [42].

### 3.3. Gait Outcomes

Appendix B describes the outcome measures of each study including stride length, step length, step width, cadence, single limb support, and double limb support. Within the studies containing a stroke population, there were five studies involving changes in stride length after treadmill training [14,15,18,25,28]. There were eight PD studies involving changes in stride length [33,36,37,38,40,41,42,44]. Out of the total 32 studies, 40.63% of the studies mentioned stride length. As for step length, there were 12 stroke studies measuring changes in step length [15,17,18,19,20,22,23,24,26,29,30,31], and 6 PD studies [34,35,36,43,44,45], which make up 56.7% of the studies. Cadence was also another common spatiotemporal outcome measure amongst the studies. Nine stroke studies [14,15,16,18,20,22,24,27,32] and eight PD studies [33,34,36,38,39,40,41,42] measured cadence before and after treadmill intervention. Like step length, cadence was mentioned in 56.7% of the total 32 studies. Contrastingly, step width was not as common. Only two stroke studies [22,25] and three PD studies [35,36,40] mentioned changes in step width, which makes up 16.7% of the total studies. Only one study measured stride cycle [37].

### 3.4. Meta-Analysis

#### 3.4.1. Meta-Analysis in Stroke

**Figure 2:**
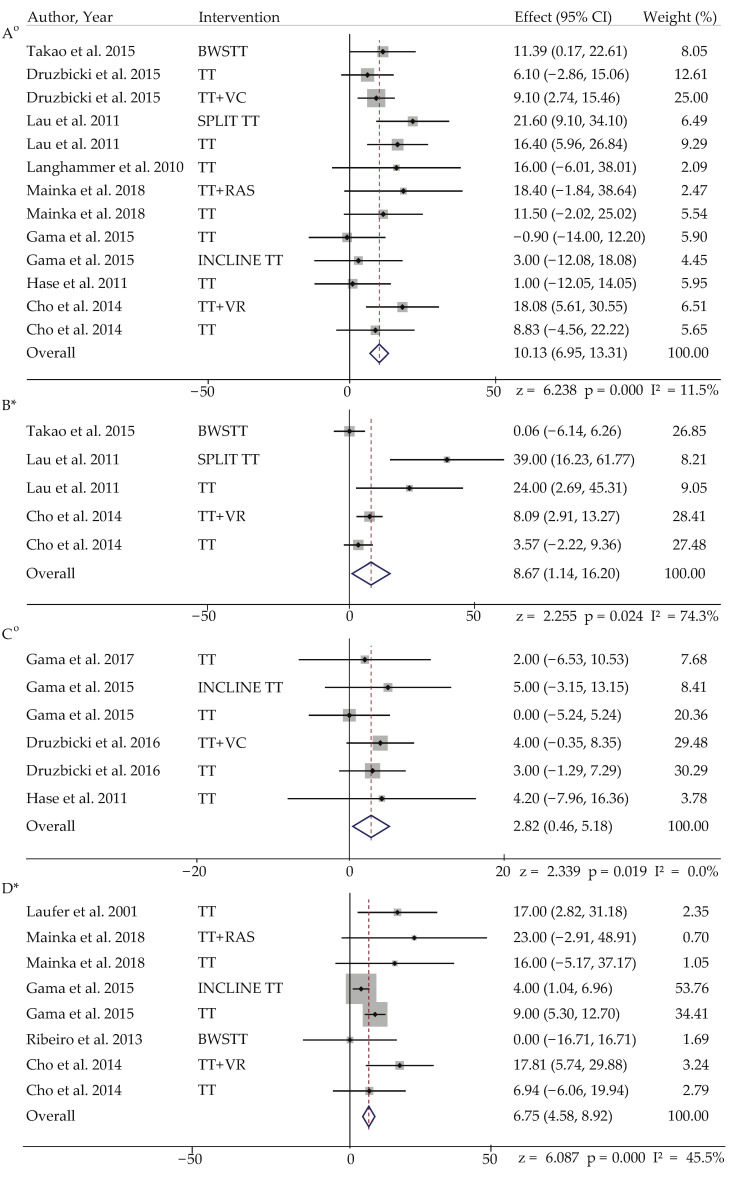
Forest plot of stroke: (**A**) cadence; (**B**) step length; (**C**) step length paretic; (**D**) stride length. Note: *—random effect model; °—fixed effect model. Abbreviations: TT: treadmill training, BWSTT: body weight support treadmill training, SpTT: speed treadmill training, IncTT: incline treadmill training, TT + VC: treadmill training with visual cues, TT + VR: treadmill training with virtual reality cues, TT + RAS: treadmill training with rhythmic auditory stimulation.

For the overall meta-analysis, the strongest effect was found in cadence after TT with visual cues and pure TT (z = 6.24, *p* < 0.001, W = 25%). In addition, TT on incline surface (W = 53.76%) and pure TT (W = 40.60%) had the most significant effect and highest weightage on stride length in adults with stroke (z = 6.08, *p* < 0.001). Interestingly, step length in paretic leg also showed significant improvements after TT with visual cues (W = 29.48%) and pure TT (W = 60.40%) had the highest weightage (z = 2.34, *p* < 0.05) in comparison with step length on non-paretic length, which was not significant (z = 0.29, *p* > 0.05). Additionally, there was significant difference in step length after TT, including virtual reality (W = 28.41%) and pure TT (W = 36.53%), which had the highest weightage (z = 2.25, *p* < 0.05). Lastly, we had one group of non-treadmill interventions in people with stroke and found prosthetic walking to have the strongest effect on cadence, though it was not significant (z = 0.90, *p* > 0.05) (Table 2, Figure 2).

#### 3.4.2. Meta-Analysis in PD

**Figure 3:**
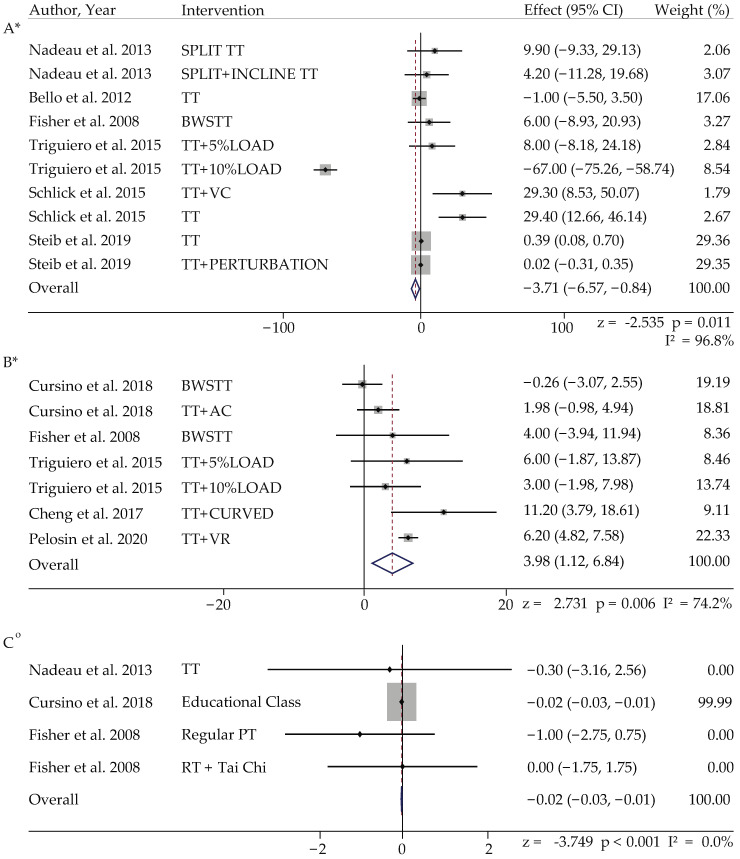
Forest plot of Parkinson’s disease: (**A**) stride length; (**B**) step length; (**C**) step width. Note: *—random effect model; °—fixed effect model. Abbreviations: TT: treadmill training, BWSTT: body weight support treadmill training, TT + (C path): treadmill training with a curved path, SpTT: speed treadmill training, IncTT: incline treadmill training, TT + VC: treadmill training with visual cues, TT + AC: treadmill training with auditory cues, TT + 5% L: treadmill training with 5% load, TT + 10% L: treadmill training with 10% load, TT + P: treadmill training with perturbations.

For the overall meta-analysis of patients with PD, TT with virtual reality (W = 22.33%) and body weight support treadmill training (W = 49.75%) had the highest weightage (W) and strongest effect on step length in adults with PD (z = 2.73, *p* < 0.05). In addition, pure TT, TT with auditory cues and body support TT also showed weightage and effect on cadence and step width in adults with PD, but this effect was not significant (cadence; z = 0.99, step width; z = 0.37, *p* > 0.05). In contrast, TT with either perturbation training (W = 29.35%) or pure TT (W = 49.09%) had the highest weightage and smallest effect on stride length in adults with PD (z = −2.53, *p* < 0.05) (Table 3, Figure 3).

#### 3.4.3. Meta-Analysis in Active Control of PD Population

We only found one significant effect of TT intervention on step width (pure TT: z = −3.74, *p* = 0.001, W = 99.90%); other than that, all effects of non-treadmill interventions on cadence, step length, and stride length were not significant (Table 3, Figure 3).

### 3.5. Publication Bias

**Figure 4:**
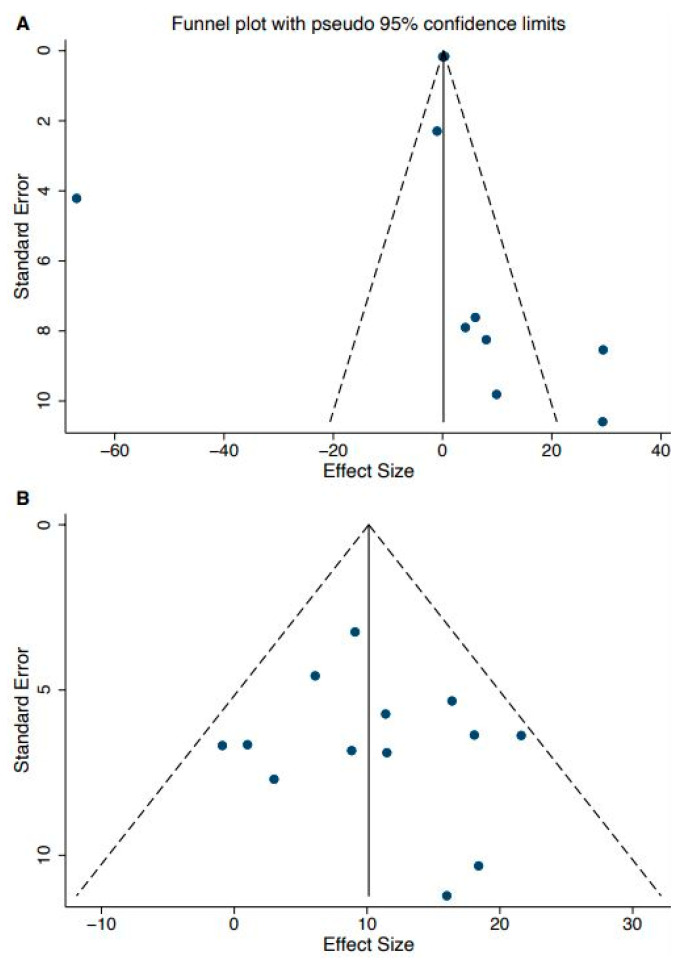
Funnel plot of Parkinson’s disease population—stride length (**A**), and stroke population—cadence (**B**).

Lastly, we performed a publication bias analysis on the cadence and stride length effect sizes in adults with stroke and PD due to the higher number of studies in these groups. Egger’s test indicated no presence of publication bias across all intervention studies, including measures of cadence (*n* = 13, *p* = 0.62) or stride length (*n* = 10, *p* = 0.69) (Figure 4).

### 3.6. Study Quality Assessment

Studies included in the current meta-analysis averaged a score of 16.55 out of 20 possible points. The distribution of the scores depended on the category of criteria [13]. All the studies were randomized controlled trials, specified the study population, had valid and consistent dependent and independent variables, and defined gait cycle parameters [14,15,16,17,18,19,20,21,22,23,24,25,26,27,28,29,30,31,32,33,34,35,36,37,38,39,40,41,42,43,44,45]. In contrast, only 12 studies provided sample size justification, or a power description [14,15,20,21,22,26,28,29,34,42,43,44], and 13 studies mentioned confounding variables and statistical adjustments [16,19,21,24,26,28,29,35,36,40,42,43,44]. Of 32 studies, 30 included an active control group [14,15,16,17,18,19,20,21,22,23,24,25,26,27,28,31,32,33,34,35,37,38,39,40,41,42,43,44,45,46] and 21 out of 32 matched their baseline sample size groups by age and gender [15,16,17,19,20,21,23,24,26,28,29,33,35,36,37,38,40,41,43,44,45]. Lastly, only 13 out of 32 studies mentioned adjustments for confounding variables in their statistical analyses [16,19,21,24,26,28,29,35,36,40,42,43,44] (Table 4).

## 4. Discussion

This study systematically reviewed and quantitatively synthesized existing scientific evidence on TT intervention studies among adults with and without PD or stroke. Gait outcome measures, including cadence, step length, and stride length of adults with neurological disorders, were examined in 30 published studies. This systematic review explicitly targeted: (1) quantifying the different treadmill interventions effects on gait parameters in different populations; and (2) evaluating each study based on a pre-defined set of quality assessment criteria.

Results from our meta-analysis show that overall TT interventions had the largest effect on cadence, step length, and stride length in adults with stroke and PD. Pure TT results are similar to previous reviews showing that task-specific TT had the greatest effect on spatiotemporal gait parameters, especially stride length and step length in people with PD [46]. Furthermore, TT with sensory feedback such as virtual and auditory feedback had greater effects on gait; these findings are similar to Baram et al., who also reported that sensory feedback with TT had larger effects on gait speed and stride length in adults with PD [47]. BWSTT results were also similar to other reviews conducted in adults with PD to improve gait [48,49]; this training is safe, successful, and complementary to therapies such as repetitive transcranial magnetic stimulation, visual cues, or transcranial direct current stimulation in adults with PD [48]. We also found improvement in gait kinematics in people with PD; however, those effects were larger in adults with stroke. Lastly, in adults with stroke, cadence, stride length, and step length on paretic leg relative to step length on non-paretic leg had the largest effect after treadmill training with visual cues, pure treadmill training, and incline treadmill training.

### 4.1. Pure Treadmill Training

Neurorehabilitation TT programs are goal-based, repetitive, and include intensive motor learning components for adults with or without PD and stroke. From our systematic review and meta-analysis, it can be seen that pure TT increases stride length in adults with PD [25,33,41], and step length in adults with stroke [22], which can be beneficial in helping them to develop a compensatory strategy for everyday activities.

### 4.2. Treadmill Training with an Incline or Speed-Dependent Treadmill Training

Speed-dependent treadmill training is a repetitive, intensive TT in which the belt speed is increased or decreased incrementally by 10% based on the individual’s performance. Incline-based TT is a repetitive, intensive training in which incline level is increased incrementally with the belt speed based on the performance of individuals. The results from our systematic review indicate that speed-dependent TT was more effective to increase stride length and gait speed in individuals with stroke in comparison with pure TT [23]. Speed- and incline-dependent (mixed) TT were also effective in improving gait speed, cadence, and stride length for patients with PD [40]. A likely rationale behind the effectiveness of incline/speed-dependent TT is that walking on inclined surfaces increases lower extremity muscle activity and may be an excellent means to improve endurance and strength. Furthermore, using inclined surfaces also decreases the monotony associated with repeated TT programs by varying the type of stimulus received in sessions [40]. After these studies, adults were successful in improving their quality of life and decreasing gait-freezing episodes, which further highlights the significance of implementing mixed TT programs

### 4.3. Treadmill Training with Sensory Feedback

Treadmill training with sensory feedback is a task involving training on a treadmill while providing sensory feedback such as auditory cues, visual cues, rhythmic cues, or a combination of these. From our systematic review, we found that auditory cues had the strongest effect on step width and step length in adults with PD [35]. We also found improvements in cadence after TT with rhythmic auditory stimulation in adults with stroke [26]. TT with auditory stimulation from functional music leads to greater improvement in functional gait. This could be due to an increased symmetry in movement with music tempo feedback [26].

Moreover, in adults with stroke, we found that visual cues with treadmill training have the strongest effect on cadence, paretic step length, and non-paretic step length [16,17]. There was an improvement in motivation due to an increase in the control of movement among these adults, because they can visualize the accuracy of the task on the screen during the training [17,50]. Visual biofeedback is useful in training patients post-stroke because it provides information about the accuracy and performance of tasks. Visual feedback provides an effective way to alter gait patterns and improve the frequency of steps, symmetry, and the coordination of gait in people with stroke [14,16].

Given that gait is a complex sensorimotor behavior that involves the coordination of neural networks, bones, muscles, and joints, it is not surprising that sensory information can aid and even influence gait performance [51]. Work from Mahoney and colleagues examining healthy older adults demonstrated that the successful ability to integrate concurrent visual and somatosensory information is associated with faster gait velocity, longer strides, a smaller percentage of the gait cycle spent in double support, and less stride length variability compared with those with unsuccessful multisensory integration abilities [52,53].

### 4.4. Treadmill Training with Bodyweight Support

Bodyweight support TT is locomotor training on a treadmill with partial body weight supported (PBWS) with an overhead harness, a pelvic belt, and thigh straps. PBWS is effective in improving mobility outcomes in adults with stroke and spinal cord injuries [52]. The load percentage in PBWS also has additional gains in improving gait functionality [53].

From our systematic review, we found that TT with PBWS can create better gait kinematics, symmetry, velocity, and endurance [27,28]. PBWS is beneficial for overground gait as well as patients with PD [25]. Ribiero et al. also found that TT with PBWS can also increase gait speed and step length in adults with stroke [29]. It was shown that TT with PBWS was able to activate central pattern generators in the spinal cord, which produces constant rhythm in walking in post-stroke survivors, thus helping to increase measures such as step length and cadence [31]. TT with PBWS is not only effective in people with stroke, but it is also effective in improving mobility in people with PD, although the effects appeared to be only short-term [39]. Fortunately, people with PD were able to tolerate a higher treadmill speed. In contradiction, Trigueiro et al. did not find any differences based on the PBWS weight load in people with PD [44], which may be attributable to the small sample size. Surprisingly, there were fewer studies using TT with PBWS as an intervention in people with PD.

### 4.5. Other Treadmill training: Curved TT and Perturbation TT

Curved walking involves the coordination of whole-body movement and the complex integration of multiple sensory systems and motor output to respond to balance demands [34]. Curved walking improves balance and speed compared with straight walking and has been effective in improving gait speed (by increasing step length) for people with PD. Perturbation treadmill training involves slips and trips while walking on a treadmill. Perturbation TT showed a significant increase in overground walking speed and gait stability in people with PD. It also reduces variability in step length, stride time, double limb support, and cadence [38], and stride length, stance, and swing time [43].

### 4.6. Biomechanical and Physiological Mechanisms behind Impact of TT in Adults with PD and Stroke

In adults with PD, TT improves functional capacity, balance, and quality of life after even short-term treadmill training [54]. TT has an impact on the overground walking economy [55]. TT also improves turning performance, the strength of lower limbs, and the sensory organization balance of individuals with PD [56]. Some of the biochemical effects of TT on PD are a decrease in inflammatory status, memory improvements through an increase in brain-derived neurotrophic factor levels, a reduction in stress hormone levels, and a decrease in neurodegeneration through the stimulation of neural plasticity [57].

In adults with stroke, TT has been shown to improve lower limb function and gait performance, which in turn promotes stroke recovery by inducing further brain plasticity through activation of the sensorimotor cortex, supplementary motor cortex, and cingulate motor area [58]. Furthermore, TT improves the fitness reserve by increasing peak VO_2_ levels while lowering the energy cost needed for hemiplegic gait and further enhancing motor functions [59]. Recent studies have shown that bi-hemispheric activation happens in adults with stroke through exercise training, which further increases motor recovery [60]. Lastly, aerobic training has been shown to decrease weight, body mass index, blood pressure, total cholesterol, and triglycerides in adults with stroke [60].

Although we do see these changes at physiological and biomechanical levels in adults with stroke and PD, these changes depend on the intensity and duration of exercise in each population. Through this review, we tried to show the importance of treadmill training in different neurological populations by its ability to improve specific spatial–temporal gait parameters. We were successful in showing the impact of treadmill training on spatial–temporal gait parameters. We found that although treadmill training is effective on its own, the use of sensory stimulation in unison with TT provides a more effective therapy for neurological populations, such as PD and stroke.

### 4.7. Adverse Effects of Treadmill Training

While the majority of TT studies did not report dropouts, 7/32 studies reported drop-outs due to personal reasons (e.g., problems with transport, or acute symptom or injury not directly attributable to intervention), while only a single study reported subjects dropping out due to leg pain, fear of falling, or subjective intolerance to training.

### 4.8. Study Limitations

We found changes in gait outcomes among older adults with and without neuro-logical disorders. Unfortunately, there were not enough healthy older adult studies to include them in this meta-analysis. Although no publication bias was detected using Egger’s test in adults with PD and stroke, the small number of included studies here does limit the power of the test to detect bias. The current meta-analyses could have benefited from the inclusion of additional studies to prove the results of the subgroup analysis of TT with BWS. Future work should examine the effects of these promising TT interventions on spatiotemporal gait parameters with larger sample sizes in more diverse neurological populations. Additionally, healthy older adult studies are needed to compare and improve patterns across older adults with and without neurological disorders.

## 5. Conclusions

The current review and meta-analytic study provides comprehensive information on the effect of treadmill interventions such as TT with sensory feedback, bodyweight support TT, TT on an inclined surface, and pure TT on spatiotemporal gait characteristics. Among these interventions, TT with sensory feedback or bodyweight support had the largest effect on cadence, stride length, and step length in adults with PD. Furthermore, in adults with stroke, pure TT and TT with visual cues or incline were shown to have the largest effect on step length, stride length, and cadence. Overall, TT interventions had the largest effect on cadence, step length, and stride length among adults with stroke and PD. However, due to a lack of studies in other populations, we were unable to justify the effect of all interventions across a wider number of neurological disorders. Nonetheless, this study will aid clinicians in choosing tailored interventions based on the specific needs of patients, as it provides information regarding which type of treadmill intervention program will have the largest effect on a specific gait parameter in adults with stroke and PD.

## Figures and Tables

**Table 1:** Study and participant characteristics.

Study ID	Author (Year)	Age (Mean ± SD (y))		Sample Size		
**Stroke**						
		**Int**	**Control**	**Int**	**Control**	**Type**
1	Brasileiro et al. (2015) [14]	52.4 ± 5.9 (Exp 1) 58.8 ± 7.9 (Exp 2)	57.9 ± 4.9	10 10	10	C, H and I
2	Cho et al. (2014) ^†^ [15]	65.86 ± 5.73	63.53 ± 5.54	30	15	C
3	Druzbicki et al. (2015) ^†^ [16]	59.8 ± 11.7	61.9 ± 11.4	25	25	C, I
4	Druzbicki et al. (2016) [17]	61.9 ± 11.4	59.8 ± 11.7	15	15	NR
5	Gama et al. (2015) ^†^ [18]	52.92 ± 9.51	57.64 ± 8.15	14	14	C, H and I
6	Gama et al. (2017) ^†^ [19]	58.7 ± 8.4	57.7 ± 10.1	14	14	C, H and I
7	Hase et al. (2011) ^†^ [20]	62.3 ± 9.2	60.1 ± 13.0	11	11	C, H and I
8	Hornby et al. (2008) [21]	57 ± 10	57 ± 11	24	24	C, H and I
9	Langhammer et al. (2010) ^†^ [22]	74 ± 13.3	75 ± 10.4	18	16	NR
10	Lau et al. (2011) ^†^ [23]	69.5 ± 11.1	72.1 ± 9.2	13	13	SA
11	Laufer et al. (2001) ^†^ [24]	66.6 ± 7.2	69.3 ± 8.1 68 ± 7.6 (HOA)	13	12 8	SA, H and I
12	Lura et al. (2019) [25]	63.8 ± 10.8	60.4 ± 16.1	18	20	A
13	Mainka et al. (2018) ^†^ [26]	65.6 ± 8.5	61.1 ± 8.6	13	11	NR
14	McCain et al. (2008) [27]	57.0 ± 17.6	61.6 ± 8.2	7	7	A, H and I
15	Ribeiro et al. (2013) ^†^ [28]	56.45 ± 8.31	58.33 ± 8.94	11	9	C
16	Ribeiro et al. (2017) [29]	57.0	60.0	19	19	SA, H and I
17	Shin et al. (2017) [30]	58.06 ± 6.00	58.06 ± 6.00 *	17	17 *	C, H and I
18	Takao et al. (2015) ^†^ [31]	59.1 ± 12.5	59.8 ± 6.3	10	8	C, H and I
19	Ribeiro et al. (2020) [32]	57.5 ± 11	60.0 ± 19	19	19	C, H and I
**Parkinson’s Disease**						
		**Int**	**Control**	**Int**	**Control**	**H and Y**
20	Bello et al. (2012) ^†^ [33]	59.45 ± 11.32	58 ± 9.38	11	11	1–3
21	Cheng et al. (2017) ^†^ [34]	65.8 ± 11.5	67.3 ± 6.4	12	12	1–2
22	Cursino et al. (2018) ^†^ [35]	63.29 ± 11.06	72 ± 10.52	7	7	1–3
23	Fisher et al. (2008) ^†^ [36]	64 ± 14.5	63.1 ± 11.5	10	10	1–2
24	Frazzitta et al. (2009) [37]	71 ± 8	71 ± 7	20	20	3
25	Klamroth et al. (2016) [38]	64.8 ± 10.3	64.2 ± 8.5	19	20	1–3.5
26	Miyai et al. (2002) ^†^ [39]	69.5 ± 1.9	69.8 ± 1.5	11	9	2.5–3
27	Nadeau et al. (2013) ^†^ [40]	64.0 ± 6.6 (Speed) 60.1 ± 6.8 (Mixed)	64.3 ± 5.6	12 11	11	1–2
28	Protas et al. (2005) ^†^ [41]	71.3 ± 7.4	73.7 ± 8.5	9	9	2–3
29	Schlick et al. (2015) ^†^ [42]	71.2 ± 10.9	68.9 ± 6.8	6	7	2–4
30	Steib et al. (2019) ^†^ [43]	67.8 ± 8.2	62.5 ± 7.9	18	20	1–3.5
31	Trigueiro et al. (2015) ^†^ [44]	61.44 ± 11.91 (5% weight) 63.44 ± 8.79 (10% weight)	61.89 ± 6.79	9 9	9	2–3
32	Pelosin et al. (2020) [45]	73.2 ± 3.6	71.9 ± 4.1	17	22	2–3

Notes: Values are in mean ± or as otherwise indicated; ^†^ represents studies included in meta-analysis; * indicates the control group’s specific neurological population the same as the intervention group. Abbreviations: HOA = healthy older adults, PD = Parkinson’s disease, C = chronic, A = acute, SA = subacute, I = ischemic, H = hemorrhagic, NR = not reported, Int = intervention, H and Y = Hoehn and Yahr scale.

**Table 2:** Meta-analysis in people with stroke.

Stroke					
Author, Year	Gait Parameter	Intervention	Effect Size (z), Overall Effect (95% CI)	I^2^ (%)	Weightage
Cho et al. 2014 [15]	Cadence	TT + VR	6.238 *** **10.128 (6.946 to 13.310)**	11.5	6.51
Cho et al. 2014 [15]		TT			5.65
Druzbicki et al. 2015 [16]		TT			12.61
Druzbicki et al. 2015 [16]		TT + VC			25.00 ^
Gama et al. 2015 [18]		TT			5.90
Gama et al. 2015 [18]		IncTT			4.45
Hase et al. 2011 [20]		TT			5.95
Langhammer et al. 2010 [22]		TT			2.09
Lau et al. 2011 [23]		SpTT			6.49
Lau et al. 2011 [23]		TT			9.29
Mainka et al. 2018 [26]		TT + RAS			2.47
Mainka et al. 2018 [26]		TT			5.54
Takao et al. 2015 [31]		BWSTT			8.05
Cho et al. 2014 [15]	Step Length	TT + VR	2.255 * **8.670 (1.136 to 16.203)**	74.3	28.41 ^
Cho et al. 2014 [15]		TT			27.48
Lau et al. 2011 [23]		SpTT			8.21
Lau et al. 2011 [23]		TT			9.05
Takao et al. 2015 [31]		BWSTT			26.85
Druzbicki et al. 2015 [16]	Step Length (Paretic)	TT + VC	2.339 * **2.821 (0.457 to 5.184)**	0	29.48
Druzbicki et al. 2015 [16]		TT			30.29 ^
Gama et al. 2015 [18]		IncTT			8.41
Gama et al. 2015 [18]		TT			20.36
Gama et al. 2017 [19]		TT			7.68
Hase et al. 2011 [20]		TT			3.78
Druzbicki et al. 2015 [16]	Step Length (Non-Paretic)	TT + VC	0.291 0.381 (−2.188 to 2.950)	0	30.32 ^
Druzbicki et al. 2015 [16]		TT			26.30
Gama et al. 2015 [18]		IncTT			13.29
Gama et al. 2015 [18]		TT			11.91
Gama et al. 2017 [19]		TT			7.59
Hase et al. 2011 [20]		TT			10.60
Cho et al. 2014 [15]	Stride Length	TT + VR	6.087 *** **6.748 (4.575 to 8.921)**	45.5	3.24
Cho et al. 2014 [15]		TT			2.79
Gama et al. 2015 [18]		IncTT			53.76 ^
Gama et al. 2015 [18]		TT			34.41
Laufer et al. 2001 [24]		TT			2.35
Mainka et al. 2018 [26]		TT + RAS			0.70
Mainka et al. 2018 [26]		TT			1.05
Ribeiro et al. 2013 [32]		BWSTT			1.69
Hase et al. 2011 [20]	Control—Cadence	Prosth Walking	0.900 3.658 (−4.307 to 11.623)	0	45.76 ^
Langhammer et al. 2010 [22]		Ground Walking			30.16
Takao et al. 2015 [31]		No Change			40.33

Note: For *p* values: *p* < 0.05 *; *p* < 0.001 ***; ^ highest weighted intervention for specific measure.; bold notes significant effect size. Abbreviations: TT: treadmill training, BWSTT: body weight support treadmill training, Prosth: prosthetic, SpTT: speed treadmill training, IncTT: incline treadmill training, TT + VC: treadmill training with visual cues, TT + VR: treadmill training with virtual reality cues, TT + RAS: treadmill training with rhythmic auditory stimulation.

**Table 3:** Meta-analysis in people with PD.

Parkinson’s Disease (PD)					
Author, Year	Gait Parameter	Intervention	Effect Size(z), Overall Effect (95% CI)	I^2^ (%)	Weightage
Bello et al. 2012 [33]	Cadence	TT	0.991 2.051 (−2.005 to 6.107)	59.9	18.67 ^
Cheng et al. 2017 [34]		TT + (C path)			11.25
Fisher et al. 2008 [36]		BWSTT			11.25
Miyai et al. 2002 [39]		BWSTPertuT			14.46
Nadeau et al. 2013 [40]		SpTT			7.51
Nadeau et al. 2013 [40]		SpTT + IncTT			14.09
Protas et al. 2005 [41]		TT			12.88
Schlick et al. 2015 [42]		TT + VC			4.47
Schlick et al. 2015 [42]		TT			5.42
Cursino et al. 2018 [35]	Step Width	BWSTT	0.367 0.005 (−0.022 to 0.031)	74.7	49.98 ^
Cursino et al. 2018 [35]		TT + AC			49.98 ^
Fisher et al. 2008 [36]		BWSTT			0.02
Nadeau et al. 2013 [40]		SpTT			0.01
Nadeau et al. 2013 [40]		SpTT + IncTT			0.01
Cheng et al. 2017 [34]	Step Length	TT + (C path)	2.731 ** **3.982 (1.124 to 6.839)**	74.2	9.11
Cursino et al. 2018 [35]		BWSTT			19.19
Cursino et al. 2018 [35]		TT + AC			18.81
Fisher et al. 2008 [36]		BWSTT			8.36
Trigueiro et al. 2015 [44]		TT + 5% L			8.46
Trigueiro et al. 2015 [44]		TT + 10% L			13.74
Pelosin et al. 2020 [45]		TT + VR			22.33 ^
Bello et al. 2012 [33]	Stride Length	TT	−2.535 * **−3.706 (−6.571 to −0.841)**	96.8	17.06
Fisher et al. 2008 [36]		BWSTT			3.27
Nadeau et al. 2013 [40]		SpTT			2.06
Nadeau et al. 2013 [40]		SpTT + IncTT			3.07
Schlick et al. 2015 [42]		TT + VC			1.79
Schlick et al. 2015 [42]		TT			2.67
Steib et al. 2019 [43]		TT			29.36 ^
Steib et al. 2019 [43]		TT + P			29.35
Trigueiro et al. 2015 [44]		TT + 5% L			2.84
Trigueiro et al. 2015 [44]		TT + 10% L			8.54
Bello et al. 2012 [33]	PD Control—Cadence	Ground Walking	1.293 2.390 (−1.234 to 6.015)	66.9	20.66
Cheng et al. 2017 [34]		Trunk Exercises			8.29
Fisher et al. 2008 [36]		Educational Class			11.87
Fisher et al. 2008 [36]		Regular PT			9.32
Miyai et al. 2002 [39]		Regular PT			24.76 ^
Nadeau et al. 2013 [40]		RT + Tai Chi			19.09
Protas et al. 2005 [41]		No Change			6.01
Cursino et al. 2018 [35]	PD Control—Step Width	TT	−3.749 *** **0.020 (−0.031 to −0.010)**	0	99.99 ^
Fisher et al. 2008 [36]		Educational Class			0.00
Fisher et al. 2008 [36]		Regular PT			0.00
Nadeau et al. 2013 [40]		RT + Tai Chi			0.00
Cheng et al. 2017 [34]	PD Control- Step Length	Trunk Exercises	1.855 1.024 (−0.058 to 2.106)	0	4.05
Cursino et al. 2018 [35]		TT			3.44
Fisher et al. 2008 [36]		Educational Class			1.65
Fisher et al. 2008 [36]		Regular PT			1.79
Trigueiro et al. 2015 [44]		TT			5.27
Pelosin et al. 2020 [45]		TT			83.80 ^
Bello et al. 2012 [33]	PD Control- Stride Length	Ground Walking	0.290 0.592 (−3.409 to 4.593)	0	61.94 ^
Fisher et al. 2008 [36]		Educational Class			3.94
Fisher et al. 2008 [36]		Regular PT			9.90
Nadeau et al. 2013 [40]		RT + Tai Chi			5.67
Trigueiro et al. 2015 [44]		TT			18.56

Note: For *p* values: *p* < 0.05 *; *p* < 0.01 **; *p* < 0.001 ***; ^ highest weighted intervention for specific measure.; bold notes significant effect size. Abbreviations: PT: physical therapy, TT: treadmill training, BWSTT: body weight support treadmill training, TT + (C path): treadmill training with a curved path, SpTT: speed treadmill training, IncTT: incline treadmill training, TT + VC: treadmill training with visual cues, TT + AC: treadmill training with auditory cues, TT + 5% L: treadmill training with 5% load, TT + 10% L: treadmill training with 10% load, TT + P: treadmill training with perturbations, RT: resistance training.

**Table 4:** Study quality assessment.

No.	Questions	Score
1	Was the study a randomized controlled trial?	2
2	Was the study population specified and defined?	2
3	Was sample size justification, power description, or variance and effect estimates provided?	0.79
4	Was there an active control group?	1.90
5	Was the treatment assessor or participant blinded?	1.34
6	Were the study groups baseline matched?	1.41
7	Were the exposure measures (independent variables) clearly defined, valid, reliable, and implemented consistently across all study participants?	2
8	Were the outcome measures (dependent variables) clearly defined, valid, reliable, and implemented consistently across all study participants?	2
9	Were potential confounding variables measured and adjusted statistically for their impact on the relationship between exposure(s) and outcome(s)?	1.10
10	Were the gait cycle parameters clearly defined and uniformly applied to all participants?	2
	Total (maximum score = 20)	16.55
	SD	0.45

## Data Availability

The data presented in this study are available on request from the corresponding author.

## Appendix A. Search Algorithm for Each Database

**Database**	**Key Terms, Algorithm, and Number of Articles Extracted**
PubMed/Medline (611)	((walk OR ambulatory OR mobility OR gait) AND (variability OR complexity OR unsteadiness or inconsist* OR stability OR equilibrium OR dynamics OR balance OR ataxia) AND (“neurological disorder” OR “neurological pathology” OR “multiple sclerosis” OR “Parkinson’s disease” OR “Huntington’s disease” OR ALS OR “cerebellar ataxia” OR Alzheimer OR stroke) AND (intervention OR therapy OR treatment OR “best practices”) AND (older adults OR elderly OR aged OR elder OR older OR senior OR geriatric) NOT (musculoskeletal OR posture OR postural OR animal OR robot OR amputee OR trunk OR knee OR Hip OR freezing of gait OR spasticity OR heart OR blood OR cardiac)) Refined by: LANGUAGES: (ENGLISH) Refined by: AGES: (Middle Aged: 45–64; Aged: 65+ years) Refined by: PUBLICATION DATES: (From 1 January 2000 to 31 December 2021) Total articles extracted: 611
CINAHL (125)	((walk OR ambulatory OR mobility OR gait) AND (variability OR complexity OR unsteadiness or inconsist* OR stability OR equilibrium OR dynamics OR balance OR ataxia) AND (“neurological disorder” OR “neurological pathology” OR “multiple sclerosis” OR “Parkinson’s disease” OR “Huntington’s disease” OR ALS OR “cerebellar ataxia” OR Alzheimer) AND (intervention OR therapy OR treatment OR “best practices”) AND (older adults OR elderly OR aged OR elder OR older OR senior OR geriatric) NOT (posture OR postural OR animal OR robot OR amputee OR trunk OR knee OR Hip OR freezing of gait OR spasticity OR heart OR blood OR cardiac)) Total articles extracted: 125
Scopus (732)	(walk OR ambulatory OR mobility OR gait OR locomot*) AND (variability OR complexity OR unsteadiness OR inconsist* OR stability OR equilibrium OR dynamics OR balance OR ataxia) AND ((“neurological disorder” OR “neurological pathology” OR “multiple sclerosis” OR “Parkinson’s disease” OR “Huntington’s disease” OR als OR “cerebellar ataxia” OR alzheimer)) AND ((intervention OR therapy OR treatment OR “best practices”)) AND ((older AND adults OR elderly OR aged OR elder OR older OR senior OR geriatric)) AND NOT ((posture OR postural OR animal OR robot OR amputee OR trunk OR knee OR hip OR freezing AND of AND gait OR spasticity OR heart OR blood OR cardiac OR depression)) AND (LIMIT-TO (LANGUAGE, “English”)) AND (LIMIT-TO (EXACTKEYWORD, “Gait Disorder”) OR LIMIT-TO (EXACTKEYWORD, “Elderly”) OR LIMIT-TO (EXACTKEYWORD, “Gait”)) AND (LIMIT- TO (PUBYEAR, 2021) OR LIMIT-TO (PUBYEAR, 2020) OR LIMIT- TO (PUBYEAR, 2019) OR LIMIT-TO (PUBYEAR, 2018) OR LIMIT- TO (PUBYEAR, 2017) OR LIMIT-TO (PUBYEAR, 2016) OR LIMIT- TO (PUBYEAR, 2015) OR LIMIT-TO (PUBYEAR, 2014) OR LIMIT- TO (PUBYEAR, 2013) OR LIMIT-TO (PUBYEAR, 2012) OR LIMIT- TO (PUBYEAR, 2011) OR LIMIT-TO (PUBYEAR, 2010) OR LIMIT- TO (PUBYEAR, 2009) OR LIMIT-TO (PUBYEAR, 2008) OR LIMIT- TO (PUBYEAR, 2007) OR LIMIT-TO (PUBYEAR, 2006) OR LIMIT- TO (PUBYEAR, 2005) OR LIMIT-TO (PUBYEAR, 2004) OR LIMIT- TO (PUBYEAR, 2003) OR LIMIT-TO (PUBYEAR, 2002) OR LIMIT- TO (PUBYEAR, 2001) OR LIMIT-TO (PUBYEAR, 2000)) AND (LIMIT- TO (PUBSTAGE, “final”)) AND (LIMIT-TO (DOCTYPE, “ar”)) Total articles extracted: 732
Web of Science (314)	(TS = (walk OR locomot* OR ambulatory OR mobility OR gait) AND TS = (variability OR complexity OR unsteadiness or inconsist* OR stability OR equilibrium OR dynamics OR balance OR ataxia) AND TS = (“neurological disorder” OR “neurological pathology” OR “multiple sclerosis” OR “Parkinson’s disease” OR “Huntington’s disease” OR ALS OR “cerebellar ataxia” OR Alzheimer) AND TS = (intervention OR therapy OR treatment OR “best practices”) AND TS = (older adults OR elderly OR aged OR elder OR older OR senior OR geriatric) NOT TS = (posture OR postural OR dual task OR animal OR robot OR amputee OR trunk OR knee OR Hip OR freezing of gait OR spasticity OR heart OR blood OR cardiac OR depression)) AND LANGUAGE: (English) AND DOCUMENT TYPES: (Article) Timespan: 2000–2021. Indexes: SCI-EXPANDED. Total articles extracted: 314

## Appendix B

**Table A1:** Treadmill Intervention Outcome Measures.

**Study ID**	**Outcome Measure**	**Stroke Control**		**Stroke Intervention**	
		**Pre**	**Post**	**Pre**	**Post**
1	Stride length	0.72 ± 0.17	0.79 ± 0.18	0.82 ± 0.25 (VF)	0.86 ± 0.28 (VF)
				0.60 ± 0.19 (AF)	0.70 ± 0.23 (AF)
	Cadence	157.0 ± 26.1	162.5 ± 29.4	162.5 ± 29.4 (VF)	159.0 ± 23.3 (VF)
				156.2 ± 29.8 (AF)	161.5 ± 37.2 (AF)
2	Step length	38.31 ± 8.31	41.88 ± 7.86	40.1 ± 6.47	48.19 ± 7.92
	Stride length	74.96 ± 18.31	81.90 ± 18.01	76.29 ± 14.99	94.10 ± 18.54
	Cadence	75.93 ± 18.13	84.76 ± 19.26	81.56 ± 19.88	99.64 ± 14.55
	SL support %	25.18 ± 5.86	28.09 ± 5.95	27.63 ± 5.27	33.14 ± 4.33
3	Cadence	67.5 ± 15.5	73.6 ± 16.8	68.9 ± 10.6	78 ± 12.3
4	Step length NP	33 ± 7	32 ± 7	31 ± 7	29 ± 6
	Step length P	25 ± 6	28 ± 6	24 ± 7	42.7 ± 14.5
5	Step length	30 ± 9	33 ± 11	38 ± 10	41 ± 9
	Stride length	67 ± 4	71 ± 4	76 ± 5	85 ± 5
	Cadence	70.1 ± 19.54	69.2 ± 15.61	67.2 ± 20.21	70.2 ± 20.51
6	Step length NP	37 ± 13	39 ± 14	35 ± 11	39 ± 14
	Step length P	39 ± 14	46 ± 11	40 ± 11	42 ± 12
	SL support % NP	36.0	37.6 ± 5.9	36.1 ± 4.6	36 ± 4.9
	SL support % P	26.0 ± 6.33	28.4 ± 4.0	25.7 ± 8.1	26.2 ± 7.6
7	Step length NP	36.6 ± 15.8	38.8 ± 13.5	43.5 ± 10.3	45.3 ± 8.5
	Step length P	38.5± 14.6	37 ± 8.1	38.5 ± 14.6	42.7 ± 14.5
	Cadence	93.1 ± 13	97.6 ± 15.1	90.7 ± 16.2	91.7 ± 15
	SL support % NP	30.5 ± 3.5	33.6 ± 3.6	31.1 ± 3.6	30.6 ± 5.1
	SL support % P	23 ± 6.9	26.9 ± 5	25.8 ± 7	25.5 ± 9.3
8	SL support %	20 ± 6.5	22 ± 7	20 ± 5.6	20 ± 5.6
9	Step length (right)	97 ± 40	110 ± 40	110 ± 20	110 ± 30
	Step length (left)	97 ± 40	92 ± 30	100 ± 20	110 ± 40
	Step width	11.3 ± 5.6	12.3 ± 5.3	7.2 ± 5.2	7.9 ± 5.3
	Cadence	99.3 ± 30.1	108.1 ± 35.1	81.6 ± 45.3	97.6 ± 24.2
10	Step length	68 ± 24	92 ± 31	65 ± 23	104 ± 35
	Cadence	39.5 ± 15	55.9 ± 12	36.9 ± 16.6	58.5 ± 15.9
11	Step length	40 ± 16	47 ± 18	36 ± 14	53 ± 22
		99 ± 12 (HOA)			
12	Step width	NR	16.32 ± 3.2	NR	16.5 ± 2.9
	Stride length	NR	75.6 ± 17.3	NR	70.9 ± 19.6
13	Step length	96 ± 26	112 ± 29	99 ± 31	122 ± 31
	Cadence	91.2 ± 19.6	102.7 ± 15.3	96.6 ± 25	115 ± 23.4
14	SL support %	NR	8.8 ± 3.9	NR	2.8 ± 4.2
15	Stride length	70 ± 20	70 ± 10	80 ± 20	80 ± 20
	DL support (s)	0.6 ± 0.2	0.6 ± 0.2	0.5 ± 0.2	0.5 ± 0.2
16	Step length NP	NR	34.62 ± 8.30	NR	36.29 ± 10.45 (VF)
				NR	36.09 ± 10.15 (RAC)
	Step length P	NR	31.50 ± 7.94	NR	33.23 ± 9.74 (VF)
				NR	34.68 ± 9.35 (RAC)
17	Step length NP (no SD)	36	36	37	37
	Step length P (no SD)	37	37	37	43
18	Step length	50 ± 11	0.01 ± 0.04	50 ± 11	0.06 ± 0.06 *
	Cadence	94.4 ± 22.8	0.93 ± 6.10	108 ± 30.6	11.39 ± 18.10 *
19	DL support (s)	0.75 ± 0.53	0.60 ± 0.36	0.64 ± 0.38	0.51 ± 0.22
**Study ID**	**Outcome Measure**	**PD Control**		**PD Intervention**	
		**Pre**	**Post**	**Pre**	**Post**
20	Stride length	136 ± 5	135 ± 7	129 ± 3	128 ± 7
	Cadence	118.8 ± 4.34	118.8 ± 4.7	117.6 ± 3.62	117.6 ± 4.34
21	Cadence	95.7 ± 12.7	96.2 ± 13.4	91.6 ± 10.0	82.7 ± 10.8
	Step length	38.0 ± 5.7	39.0 ± 7.6	38.3 ± 7.7	49.5 ± 10.6
22	Step length	59.2 ± 5.24	64.68 ± 5.88	65.83 ± 2.5 (PBWS)	65.57 ± 2.85 (PBWS)
				66.69 ± 2.73 (GAS)	68.67 ± 2.91 (GAS)
	Step width	0.11 ± 0.01	0.09 ± 0.01	0.06 ± 0.01 (PBWS)	0.08 ± 0.01 (PBWS)
				0.08 ± 0.01 (GAS)	0.07 ± 0.01 (GAS)
23	Step length	71 ± 8	71 ± 11	73 ± 10 (HI)	77 ± 8 (HI)
				68 ± 11 (LI)	72 ± 7 (LI)
	Step width	12 ± 2	11 ± 2	11 ± 2 (HI)	11 ± 2 (HI)
				10 ± 2 (LI)	10 ± 2 (LI)
	Stride length	137 ± 23	141 ± 23	148 ± 18 (HI)	154 ± 16 (HI)
				143 ± 15 (LI)	144 ± 14 (LI)
	Cadence	120.33 ± 9.26	121.09 ± 8.6	120.66 ± 10.4 (HI)	120.85 ± 8.5 (HI)
				120.57 ± 11.6 (LI)	118.94 ± 10.2 m(LI)
	DL support %	24.04 ± 6.17	21.22 ± 4.03	21.2 ± 3.35 (HI)	19.68 ± 2.58 (HI)
				19.53 ± 4.49 (LI)	19.87 ± 3.58 (LI)
24	Stride cycle (cycle/s)	0.6 ± 0.1	0.7 ± 0.1	0.6 ± 0.2	0.8 ± 0.2
25 **	Stride length	4.25	4.04	4.44	3.8
	Cadence	3.15	3.28	3.59	2.97
	DL support %	5.10	4.81	5.07	4.76
26	Cadence (steps/10 m)	22.8 ± 2.2	22.7 ± 2.0	23.4 ± 2.3	20 ± 2.1
27	Step width	8.1 ± 3.8	7.8 ± 3.0	8.8 ± 3 (STT)	8.7 ± 2.8 (STT)
				8.6 ± 3.9 (MTT)	8.2 ± 3.9 (MTT)
	Stride length	133 ± 19.4	135 ± 20.8	111.9 ± 25.9 (STT)	121.8 ± 22 (STT)
				136.4 ± 17.6 (MTT)	140.6 ± 19.4 (MTT)
	Cadence	113.7 ± 4.6	115.1 ± 4.8	109.1 ± 13.7 (STT)	110.2 ± 16 (STT)
				108.2 ± 8.7 (MTT)	111.2 ± 6.2 (MTT)
	SL support %	37.9 ± 1.8	38.2 ± 1.8	37.4 ± 3.1 (STT)	38.4 ± 2.4 (STT)
				37.5 ± 1.3 (MTT)	38.2 ± 1.2 (MTT)
	DL support %	24.4 ± 3.5	23.8 ± 3.4	25.5 ± 6.2 (STT)	23.4 ± 4.6 (STT)
				25.1 ± 2.7 (MTT)	23.7 ± 2.5 (MTT)
28	Stride length (right)	60.2 ± 13.3	60.4 ± 10.0	66.5 ± 13.7	71.1 ± 14.4
	Stride length (left)	61.0 ± 15.4	60.8 ± 10.9	68.7 ± 14.9	72.9 ± 17.0
	Cadence	117.7 ± 13.0	124.3 ± 15.1	112.8 ± 7.2	120.3 ± 8.2
29	Stride length	75.1 ± 18.2	104.5 ± 21.7	61.1 ± 29.6	90.4 ± 21.7
	Cadence	107.4 ± 21.8	110 ± 13.2	99.9 ± 28.1	95.4 ± 10.9
30 *	Step length	0	1.15	0	−1.85
	DL support %	0	0.10	0	0.68
31	Step length	56 ± 4	57 ± 6	45 ± 9 (5% load)	51 ± 8 (5% load)
				58 ± 3 (10% load)	61 ± 7 (10% load)
	Stride length	111 ± 11	115 ± 9	93 ± 18 (5% load)	101 ± 17 (5% load)
				191 ± 4 (10% load)	124 ± 12 (10% load)
32	Step length				

Note: All data are written as mean ± SD unless otherwise indicated. * Indicates change values. ** Indicates coefficient of variation. Units of measurement are as follows: step length (cm), step width (cm), stride length (cm), cadence (steps/min). Abbreviations: NR: not reported, VF: visual feedback, AF: auditory feedback, RAC: rhythmic auditory cues, SL: single limb, DL: double limb, P: paretic, NP: non-paretic, HOA: healthy older adults, PBWS: partial body weight support group, GAS: auditory stimulus group, HI: high intensity, LI: low intensity, STT: speed treadmill training, MTT: mixed treadmill training.
